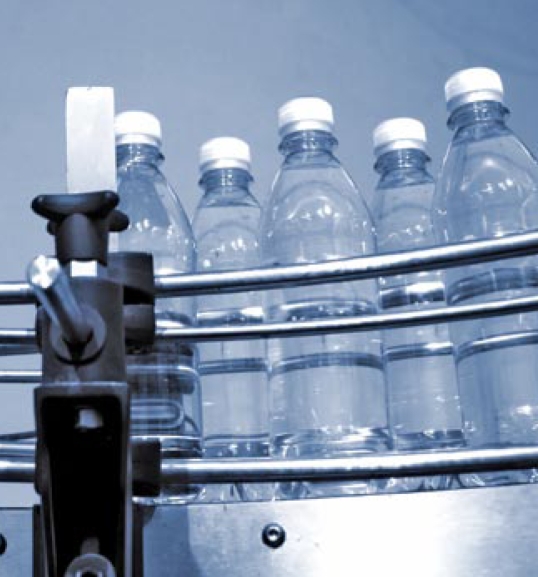# The Beat

**Published:** 2009-08

**Authors:** Erin E. Dooley

## Hearing Loss Express?

Richard Neitzel et al. report in the August 2009 *American Journal of Public Health* that New York City subways are loud enough to increase the risk for noise-induced hearing loss, an irreversible condition affecting about 250 million people worldwide. Subway platforms were the noisiest of the environments studied, with decibel levels higher than that of a chainsaw, but other forms of mass transit also were loud enough to present a risk of hearing loss with prolonged exposure. The researchers suggest that mass transit riders reduce noise exposure using earplugs or earmuffs—but not headphones or earbuds, since wearers often turn up the music to drown out ambient noise.

**Figure:**
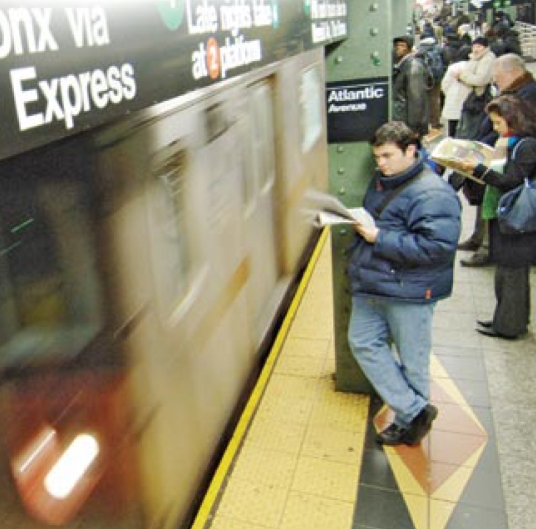


## Cell Phones Banned in French Schools

The French government has banned cell phones from primary school grounds and directed manufacturers to offer handsets that allow only text messages and phones that work only with headsets. The decision follows a six-week review of mobile phone use and wireless radiation, including reports of an association between childhood use of cell phones and increased risk of adult cancer published 4 April 2009 by Lennart Hardell et al. ahead of print in a special issue of *Pathophysiology*. But even these measures are deemed inadequate by advocates seeking a complete ban on mobile phone use by children under age 14 and limits on the power and siting of cell phone masts and towers.

## Workshops for African Medical Journal Editors

The African Journal Partnership Project (AJPP), established in 2004 by the National Library of Medicine and the Fogarty International Center, promotes capacity-building efforts for medical journals in Africa. To advance its goals, the AJPP is now planning four train-the-trainer workshops—a September 2009 workshop in Ghana will combine training for editorial and computer support staff; later workshops in Ethiopia, Uganda, and Zambia will focus on scientific writing and reviewing skills. More information about the AJPP is available at http://www.ehponline.org/international/. AJPP member *EHP* has partnered with *Mali Médical* since the project’s inception.

## Cozy Cancer Risk

The EPA’s *2002 National-Scale Air Toxics Assessment*, released in June 2009, shows that the polycyclic aromatic hydrocarbons released by wood-burning fireplaces and stoves contribute the most to the cancer risk from breathing Oregon’s air. Oregon ranked third in the nation after New York and California in the number of people living in census tracts with elevated population exposures, but this ranking may be due to Oregon’s unusually thorough documentation of wood stove and fireplace use, which is particularly popular for home heating in the western half of the state. To minimize toxic emissions, the American Lung Association recommends burning only clean, dry, seasoned hardwood and regular maintenance to prevent creosote buildup in flues.

**Figure:**
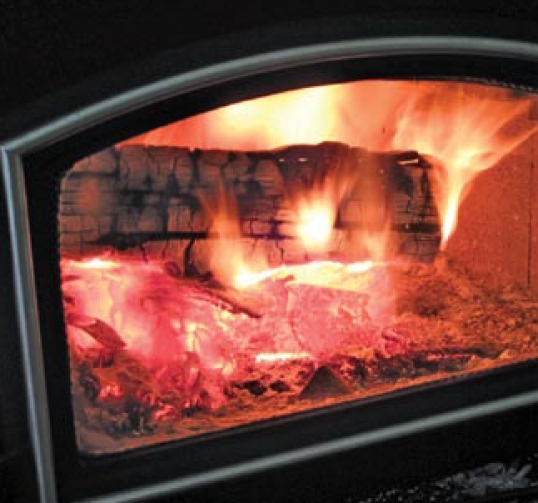


## CO Damages Fetal Rat Brain Cells

In work published 27 May 2009 in *BMC Neuroscience*, Ivan A. Lopez et al. found that chronic fetal exposure to 25 ppm carbon monoxide (CO) permanently damaged rat brain cells through oxidative stress, leading to a decrease in proteins essential for proper functioning. The authors say this exposure simulates the potential CO exposure of a human fetus whose mother is a “mild to modest” smoker. Other indoor CO exposure sources include gas appliances, fireplaces, and attached garages. There are no EPA standards for CO in indoor air.

## Better Oversight for Bottled Water

Given rising consumption of bottled water, the Government Accountability Office (GAO) evaluated the extent and strength of FDA regulations for this product. In June 2009, the GAO reported that FDA standards generally reflect those of the EPA except in the case of di(2-ethylhexyl) phthalate, a compound used to make plastic bottles, for which the FDA has no standard. The GAO recommended that the FDA issue a standard or publish reasons for not doing so, and that water bottlers provide more labeling information for consumers on the quality and safety of their products. Currently, companies (unlike water utilities) are not required to disclose positive tests for contaminants.

**Figure:**